# Patient-specific structural connectomic differences of deep brain stimulation targets in treatment-resistant obsessive-compulsive disorder patients

**DOI:** 10.1038/s41398-026-04441-4

**Published:** 2026-09-22

**Authors:** Rene Marquez-Franco, Luis Ruelas, Ricardo Loução, Rabea Schmahl, Fátima Ximena Cid Rodríguez, Petra Heiden, Jens Kuhn, Anne Koy, Veerle Visser-Vandewalle, Pablo Andrade

**Affiliations:** 1 Department of Stereotactic and Functional Neurosurgery, Faculty of Medicine, University Hospital Cologne, University of Cologne, Cologne, Germany; 2 Department of Pediatrics, Faculty of Medicine, University Hospital Cologne, University of Cologne, Cologne, Germany; 3 Center for Rare Diseases, Faculty of Medicine, University Hospital Cologne, University of Cologne, Cologne, Germany; 4 Centro de Ciencias de la Complejidad, Instituto de Ciencias Nucleares, Universidad Nacional Autónoma de México, Mexico City, Mexico; 5 Department of Psychiatry, Faculty of Medicine, University Hospital Cologne, University of Cologne, Cologne, Germany; 6 Alexianer Hospital Cologne, Alexianer Köln GmbH, Cologne, Germany

**Keywords:** Predictive markers, Psychiatric disorders

## Abstract

Deep brain stimulation (DBS) is an established therapy for treatment-resistant obsessive-compulsive disorder (trOCD), yet clinical outcomes vary. This is partly due to differential cortico-striato-thalamo-cortical networks (CSTC) engagement across stimulation in relevant anatomical regions. This study compared the structural and functional connectivity profiles of three relevant anatomical-regions that have also served as target for DBS in different centers for trOCD: the nucleus accumbens/anterior-limb of the internal-capsule (NAc/ALIC), the medial forebrain bundle (MFB), and the anteromedial subthalamic nucleus (amSTN). We analysed in a retrospective, observational, single-center cohort the structural connectivity on patient-specific space, using structural and diffusion MRI data from 22 trOCD patients treated with NAc/ALIC DBS at the University Hospital Cologne. For this end, Whole-brain probabilistic-tractography using single-shell-three-tissue constrained-spherical-deconvolution was applied to reconstruct patient-specific structural connectivity matrices. From whole-brain tractograms, target-specific streamline subsets were selected using ROI-based inclusion criteria using 2 mm radius spheric stereotactic ROIs representing clinically relevant trOCD-DBS target regions. Afterwards, Structural connectivity matrices were extracted using Spherical-deconvolution informed filtering of tractograms (SIFT) streamline count, fractional anisotropy (FA), and mean diffusivity (MD) across 13 cortico-subcortical regions implicated in OCD pathophysiology. Thereafter, we characterized each target’s connectivity to key OCD functional networks derived from meta-analytic functional connectivity maps. NAc/ALIC demonstrated stronger structural fronto-limbic connectivity: medial-orbitofrontal cortex, anterior-cingulate, insula, and accumbens (*p* < 0.0001), associated with the affect (AN), salience (SN), default-mode (DMN), and reward-motivation (RMN) networks anatomical hubs. The MFB predominantly connected with reward-related regions: pallidum and rostral-middle-frontal cortex (*p* < 0.0001). The amSTN showed to be mainly connected to SN and the cognitive/motor control network (CMCN): precentral and paracentral gyri (*p* < 0.0001). These exploratory findings suggest that clinically relevant trOCD-DBS targets may engage distinct but partially overlapping tractography derived structural networks and meta-analytic functional networks. The observed connectivity profiles may help generate hypotheses about symptom-specific networks for future DBS planning. Overall, these results support the proof-of-concept value of patient-specific probabilistic tractography in trOCD. However, prospective validation in independent, multicentric trials remains necessary before clinical translation.

**Figure:**
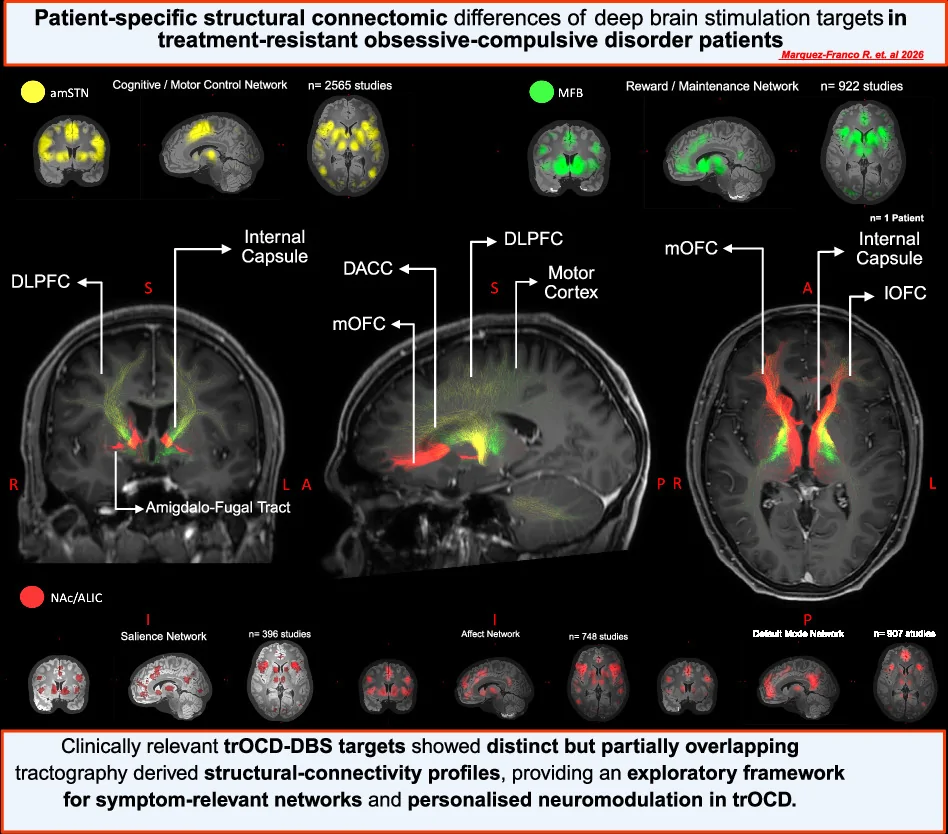

## Introduction

Obsessive-compulsive disorder (OCD) is a debilitating psychiatric disorder characterized by intrusive thoughts and repetitive, compulsive behaviors [1]. OCD patients display a considerable clinical heterogeneity of symptoms, including contamination/washing, symmetry/order, intrusive/taboo thoughts, and checking behaviors, reflecting distinct yet overlapping neurocognitive profiles [2]. These symptoms can significantly impair daily functioning and quality of life [1]. Dysfunction of the neural circuits linking the frontal cortex, striatum, thalamus, and primary and supplementary cortex is believed to underlie the repetitive and intrusive nature of obsessions and motor components of the compulsions [2]. While first-line cognitive behavioral therapy and pharmacological interventions provide relief for many patients, up to 30% remain treatment-resistant (trOCD) [2, 3]. Treatment-resistant is defined as an insufficient response to at least the following: two trials of selective serotonin-reuptake inhibitors at a maximum tolerated dose for at least 12 weeks; one trial of clomipramine at a maximum tolerated dosage for at least 12 weeks; one augmentation trial with an antipsychotic for at least eight weeks in combination with one of the aforementioned drugs; and one complete trial of exposure-based CBT confirmed by a psychotherapist [4]

Increasing evidence suggests that OCD symptom heterogeneity reflects dysfunctions across distinct large-scale brain functional networks, including the reward/maintenance network (RMN), affect network (AN), Salience network (SN), cognitive/motor control network (CMCN), and default mode network (DMN) [2]. Neuroimaging meta-analyses and functional connectivity studies have revealed that OCD is associated with abnormal engagement of cortico-striato-thalamo-cortical (CSTC) networks, as well as widespread dysfunctions of these distributed functional connectivity [2, 5]. The relevance of these networks in OCD lies in its integral role in habit formation, sensorimotor gating, and inhibitory control, functions that may underlie patients’ difficulties in suppressing intrusive thoughts and repetitive behaviours [3, 6]. Brennan et al. suggest that OCD symptoms arise from disruptions in large-scale cortical networks beyond the traditional CSTC model [6]. In trOCD cases, deep brain stimulation (DBS) has been applied as a therapeutic strategy, targeting key hubs of the CSTC circuit implicated in OCD pathophysiology [4, 7, 8]. Notably, structural and functional alterations have been consistently observed in key regions within this circuit in OCD patients, including the amygdala, nucleus accumbens, medial and lateral orbitofrontal cortices, rostral anterior cingulate cortex, insula, pallidum, rostral middle frontal cortex, precentral, paracentral, and superior frontal gyrus [9]. These areas contribute to the dysregulated integration of affective, motor, and executive functions observed in OCD patients [9, 10]. Understanding the profiles of structural and functional connectivity between these regions is essential to elucidate neurobiological hub regions related to specific symptoms [9]. Computational neurology and psychiatry research aims to adapt neuromodulatory treatment strategies such as DBS and repetitive transcranial magnetic stimulation (rTMS) [6]. In the emerging field of computational psychiatry, there is an increased focus on mapping brain connectomics to explain individual variability in emotional dysregulation, motor inhibition, and reward processing [11]. This remains an evolving area of research.

Over the past two decades, several anatomical regions for DBS targets have been clinically investigated for OCD, including the nucleus accumbens and anterior limb of the internal capsule (NAc/ALIC), the medial forebrain bundle (MFB), and the anteromedial subthalamic nucleus (amSTN), among others [7, 8, 10, 12–14]. While all have demonstrated therapeutic clinical benefits measured with the Yale-Brown Obsessive Compulsive Scale (Y-BOCS), response rates remain variable, and the neurobiological mechanisms underlying this heterogeneity are not yet fully understood [9]. Recent advances in diffusion weighted imaging and probabilistic tractography have enabled a more detailed characterization of the structural networks engaged by different anatomical regions used as DBS targets [15]. This could allow the possibility to analyse how distinct symptom dimensions may be modulated by different anatomical regions, related to specific functional networks [2, 3, 5, 6]. Contamination and washing behaviours have been associated with alterations in attention and salience-related regions, such as the superior parietal lobule, dorsolateral prefrontal cortex, and anterior insula, converging on the ventral attention and frontoparietal networks implied in CMCN [6, 11]. In contrast, harm and checking symptoms are more strongly associated with dysfunction in the DMN and AN, particularly the orbitofrontal and ventromedial prefrontal cortices, which are involved in fear learning, guilt, and safety signalling. Together, these findings highlight that OCD symptom dimensions map onto distinct functional and structural networks, supporting the need for network-based approaches to tailor therapeutic interventions [6, 11].

Normative connectome-based studies have suggested the existence of a structural common pathway associated with symptom relief measured by the clinimetric scale Y-BOCS in OCD, yet evidence points to multiple anatomical targets within the CSTC loop for the improvement of different phenotypes and symptoms of OCD not measured by Y-BOCS [11]. The conventional approach to diffusion MRI (dMRI) and functional MRI (fMRI) analysis uses anatomy of population-based templates (average brain) to define regions of interest (ROIs) in the brain. Although macroscale group-level analyses can provide valuable information about common pathways of brain organisation, they may obscure clinically relevant interindividual variability in structural and functional connectivity [11]. This is particularly important in DBS-connectomics studies, where group averaging may reduce sensitivity to patient-specific diffusion and structural MRI features that are relevant for individualised network characterisation, in turn important for clinical decision-making [15]. Consequently, the absence of patient-specific diffusion and structural MRI analyses in previous group-average studies may have masked both biologically and clinically important discrepancies [6, 11].

On the other hand, patient-specific anatomically constrained probabilistic tractography offers a promising approach to quantify the structural connectivity profiles of individual patients, which takes into account the individual variability in white matter [15]. In this study, we aim to characterize and compare the tractography derived structural connectivity profiles of three clinically relevant trOCD DBS targets: NAc/ALIC, MFB, and amSTN, using patient-specific diffusion tractography in the same individual hemisphere in trOCD patients. We hypothesize that each target has different remote structural connectivity that engages distinct functional networks, reflecting their potential to differentially modulate affective, cognitive, or motor symptom domains in trOCD patients.

## Materials and methods

### Patient Cohort

In this retrospective, observational, single-center cohort study, we analysed structural connectivity using patient-specific diffusion and structural MRI data in native space. Each clinically relevant DBS target for trOCD (NAc/ALIC, MFB and amSTN) was represented by a standardised spherical ROI with a radius of 2 mm. We analyzed structural (T1 and T2-MRI) and diffusion-weighted imaging (DWI) data from twenty-two trOCD patients who underwent NAc/ALIC DBS surgery between 2016 and 2025 at the Department of Stereotactic and Functional Neurosurgery of the University Hospital Cologne. Inclusion criteria included a confirmed DSM-5 diagnosis of OCD and disease severity measured by Y-BOCS, and treatment-resistant as defined in [4, 16]. Exclusion criteria for this study included a history of neurological disease and incomplete MRI sequence data. Additional methodological details are provided in the Supplementary Material.

### Ethical considerations

All procedures were approved by the Ethics Committee of the University of Cologne and adhered to the principles of the Declaration of Helsinki.

### Imaging acquisition

MRI was performed using a 3 T system (Philips Ingenia, Eindhoven, Netherlands). T1- and T2-weighted structural images were acquired with 0.5 ×0.5 ×1 mm³ resolution. T1 with TR/TE: 9.75/4.86 ms, and T2 with TR/TE: 3000/80 ms. Diffusion-weighted images (dMRI) included 40 gradient directions (b = 1000 s/mm²) with AP phase encoded direction, 1 b0 with AP phase encode, and 1 b0 with PA phase encode. dMRI with TR/TE: 8218/103 ms. dMRI resolution was 2 mm isotropic. Each scanning session lasted approximately 20 minutes.

### MRI preprocessing

We implemented a pipeline designed to perform probabilistic tractography and structural connectome generation in patient-specific space. The pipeline facilitates batch processing across multiple subjects, reporting the results in patient space. Data were processed using the FMRIB Software Library FSL (version 6.07.18) [17], Freesurfer (v7.4.1) [18], Advanced Normalization Tools (ANTs v3.26.3) [19], MRtrix3 [20], lead-DBS (V.3.1) [21], and DSI-Studio [22]. For further details, please check the supplementary material section.

For preprocessing, we used the following steps: denoising, de-ringing, and correction of motion artifacts, b0-field inhomogeneity, eddy currents, and artifacts [17, 20, 23, 24, 25, 26]. White-matter, gray-matter, and cerebrospinal fluid (CSF) tissues response functions were estimated via the Dhollander algorithm, and fiber orientation distributions (FODs) were computed using single-shell, 3-tissue spherical deconvolution models [27–30]. Skull stripping and segmentation were done with FSL and Freesurfer, respectively, while ANTs were used for nonlinear registration of T1-MNI 2009bAsym, patients’ T1 and T2 to diffusion DWI space [17, 19, 31, 32]. Whole-brain probabilistic tractography was performed for each subject using MRtrix3. Anatomically constrained tractography (ACT) was used to improve the anatomical plausibility of streamline termination at the grey-white matter interface (GMWMI) [33]. For each patient, 10 million streamlines were initially generated in the whole brain. Streamline length was restricted from 10 to 250 mm, the minimum FODs amplitude threshold was set to 0.05, and the maximum angular deviation was set to 25 angular degrees. Subsequently reduced to 1 million streamlines using Spherical-deconvolution Informed Filtering of Tractograms (SIFT) [34]. SIFT was implemented to reduce known reconstruction biases and to improve the coherence between streamline density and fiber density estimated from FODs. SIFT was used as an a priori component of the processing pipeline rather than as an experimental factor. A supplementary figure 1 shows methodological quality control sensitivity comparison was performed to visualise the effect of SIFT on target-specific streamline counts; this was not treated as an experimental factor.

### trOCD targets probabilistic tractography coordinates

From the 1 million SIFT-processed whole-brain streamlines, target-specific streamline subsets were selected using ROI-based inclusion criteria implemented with “tckedit” using 2 mm radius spheric stereotactic ROIs representing clinically relevant trOCD-DBS target regions

For the comparative patient-specific analysis, each clinically relevant trOCD-DBS target was modelled as a standardised 2 mm radius spherical ROI. ROI centers were defined from predefined stereotactic coordinates in MNI152NLin2009bAsym space, based on a high-resolution 7 T ex vivo T1-weighted MRI volume, and standardised using the tenths stereotactic coordinates method referenced to the BigBrain intercomisural line (AC-PC) line [35]. The ROIs were subsequently transformed into each patient’s native T1-weighted and b0 diffusion spaces using subject-specific ANTs transformations.

To support anatomical validation in each participant, the transformed ROIs were reviewed in relation to relevant subcortical landmarks, including the nucleus accumbens (NAc), the subthalamic nucleus (STN), and the red nucleus (RN). The NAc was identified using FreeSurfer derived segmentation labels, whereas the STN and RN were assessed using subcortical atlas-based definitions [18, 36, 37]. The anatomical validity of the transformed spherical ROIs was independently evaluated by two reviewers: an experienced functional neurosurgeon (PA) and an experienced postdoctoral researcher in functional neurosurgery (RMF). Both reviewers confirmed the appropriate localisation of the stereotactic target ROIs in native patient space.

**Fig. 1:**
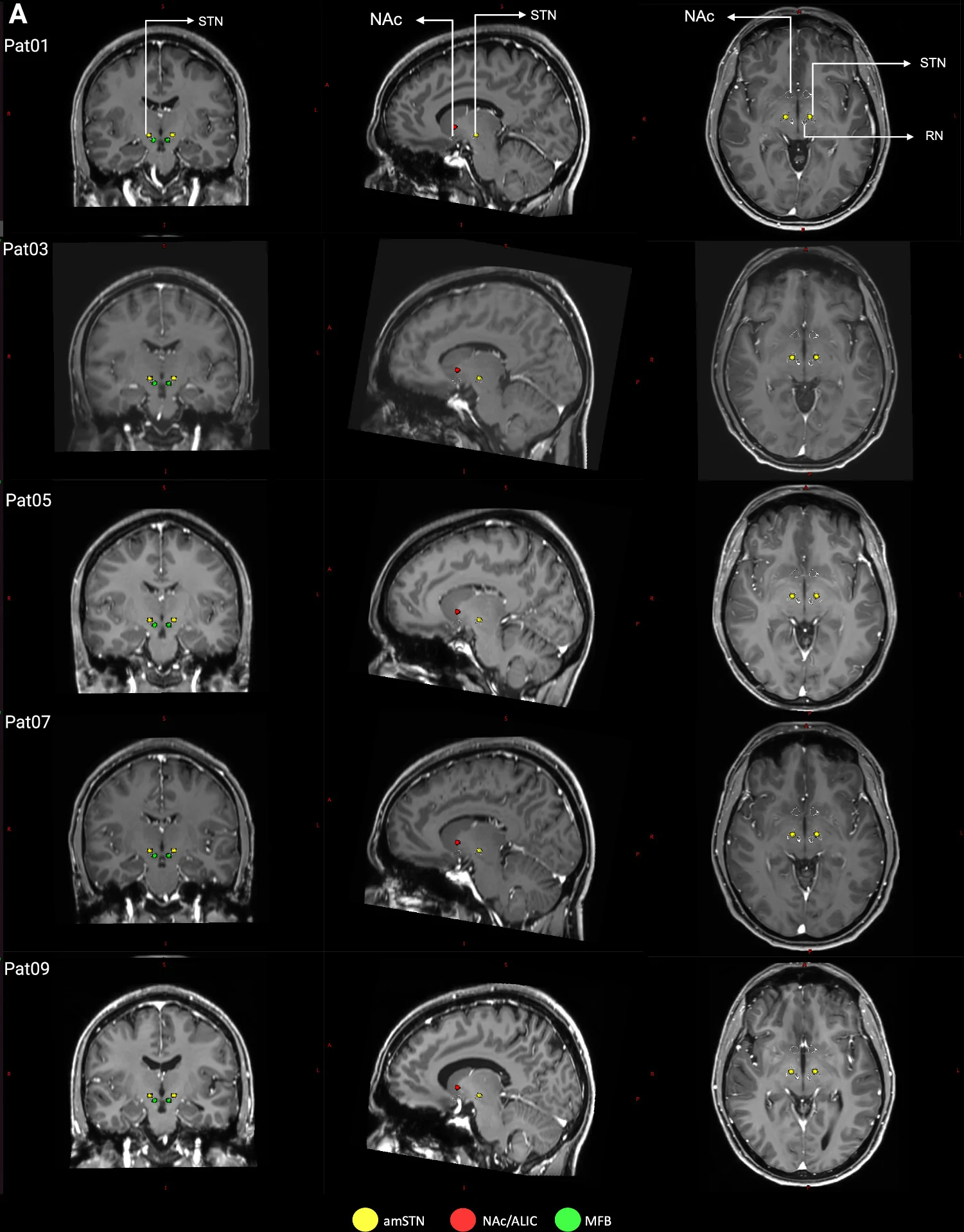

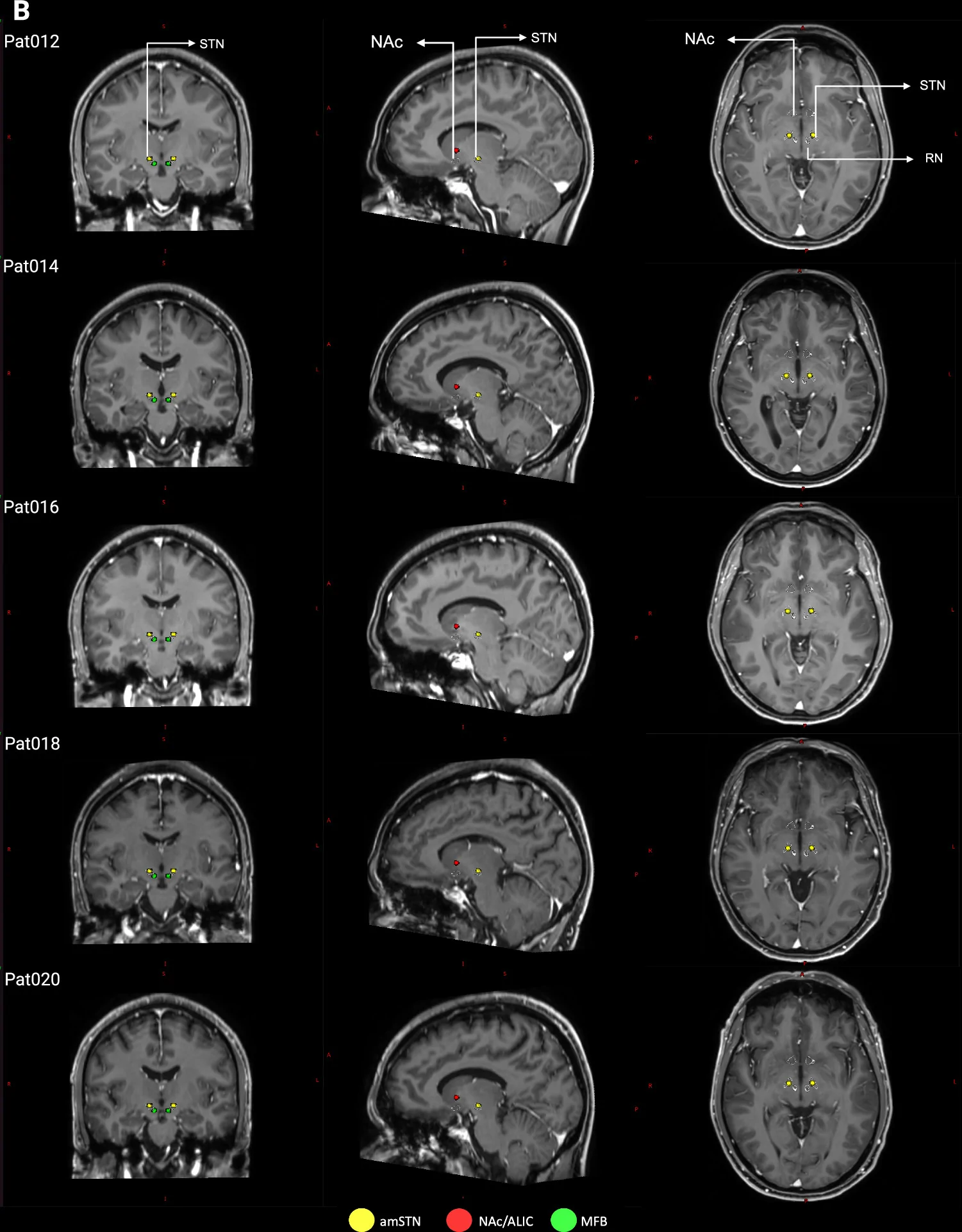
A and 1B. Patient-specific localisation of standardised spherical ROIs representing trOCD-DBS targets in native anatomical space. Native space localisation of three trOCD-relevant DBS targets, the nucleus accumbens/anterior limb of the internal capsule (NAc/ALIC, red), the anteromedial subthalamic nucleus (amSTN, yellow), and the medial forebrain bundle (MFB, green), in ten randomised participants. Figure 1**A** shows the odd-numbered participants (Pat01, Pat03, Pat05, Pat07, Pat09) and 1**B** the even-numbered participants (Pat012, Pat014, Pat016, Pat018, Pat020); each row displays coronal, sagittal, and axial views of the native T1-weighted image. Bilateral 2 mm radius spherical ROIs were centered on each participant’s patient-specific target coordinates, and anatomical annotations in the first row of each panel indicate the nucleus accumbens (NAc), subthalamic nucleus (STN), and red nucleus (RN). Identically sized ROIs standardised the spatial sampling volume across targets and participants, enabling direct comparison of the subsequent structural-connectivity analyses.

The resulting masks should be interpreted as standardised stereotactic ROIs mapped into patient space rather than as individually selected surgical targets. This strategy ensured comparable spatial sampling across targets while allowing tractography derived structural connectivity estimates to be calculated from each patient´s native diffusion data. For anatomical reference, (Fig. 1) show the bilateral NAc/ALIC, amSTN, and MFB ROIs in 10 randomised patients. To improve anatomical transparency and document the spatial consistency of the patient-specific tractography workflow, the supplementary material provides the predefined stereotactic coordinates in Supplementary Table 1, together with a detailed description of the ROI transformation procedure.

### Tract registration and analysis

To align MRtrix3 tractograms with DSI-Studio (v.2023), FA maps were used as a reference and co-registered using ANTs antsRegistration. FA and MD maps were computed from each patient´s own diffusion MRI data. After linear registration between MRtrix3 and DSI-Studio spaces, we applied a spatial transformation to the tracks files from MRtrix3 to DSI-Studio using the tcktransform algorithm. Subsequently, diffusion derived metrics were sampled along the target-specific streamline selections in DSI-Studio [38, 39]. Atlas parcelations were used for anatomical labelling and a-priori ROIs definitions. The CerebrA atlas guided the a priori ROIs in cortical and subcortical regions [40]. The HCP842_tractography atlas guided white matter bundles: meso-limbic fiber bundle, the frontopontine-thalamocortical tracts (Fp-Ct) definition [41]. The CerebrA atlas guided the a priori ROIs streamline count [40]. Metrics included a priori ROIs streamline count, FA, and MD [15, 40, 42–45]. No healthy subject diffusion metrics or normative diffusion values were used for tractometric analysis [38, 39].

### Structural and functional connectivity

We calculated the streamline count between each brain hemispheres NAc/ALIC, amSTN, and MFB tractogram and a priori ROIs (amygdala, accumbens, medial orbitofrontal cortex, lateral orbitofrontal cortex, rostral anterior cingulate, insula, rostral middle frontal, pallidum, precentral, paracentral, superior frontal cortex) and meso-limbic fiber bundle, the frontopontine-corticalthalamic tracts (Fp-Ct) based on the aforementioned atlas [39–41]. Streamline count from ROIs that passed through the NAc/ALIC, amSTN and MFB were quantified [37]. Overall, FA and MD values were averaged along each tract and compared across targets [46].

Streamline count and diffusion derived metrics were analysed separately. Streamline count was interpreted as a tractography derived estimate of relative structural connectivity, whereas FA and MD were sampled from each participant’s diffusion tensor derived metric maps along the target-specific streamline selections. For tractometry, diffusion derived metrics were sampled at 100 equidistant nodes along each target-specific tract selection in DSI-Studio [38, 39, 46]. FA and MD were interpreted descriptively and not as direct measures of axonal density, myelination, or cellular microstructure.

We conducted a search based on a single term in the platform Neurosynth to obtain a meta-analysis of the open-science studies of functional connectivity. We used the terms including the RMN, AN, SN, CMCN, and DMN based on the phisyopathology of OCD [3, 5, 6]. We obtained NiFTi images based on the z-scores from a one-way ANOVA, testing whether the proportion of studies that report activation at a given voxel differs from the rate that would be expected if activations were uniformly distributed throughout gray matter [47]. Then, we identified the anatomical parcelations of the aforementioned structural ROI to make a qualitative correlation with the functional networks meta-analysis results. Further details regarding dMRI preprocessing, probabilistic tractography pipeline, DBS targets ROI definitions, registration methodological procedures, target-specific streamline selection, and patient-specific ROI masks for quality control steps are provided in the Supplementary Material.

### Statistical analysis

Data analysis was performed using Jamovi (v2.5.2) [48, 49], Python3, and MATLAB (v24.4) [50]. Distribution normality was assessed using Kolmogorov-Smirnov tests. Depending on distribution, t-tests or Wilcoxon signed-rank tests were applied to demographic and tractometric variables. For tractometry, diffusion-derived metrics were sampled at 100 equidistant nodes along each target-specific tract selection in DSI-Studio. Kruskal-Wallis with Dunn post-hoc tests comparisons were used to examine group-level differences in a priori ROIs streamline count, FA, and MD across all clinically relevant trOCD-DBS target regions.

## Results

A total of 22 patients (44 brain hemispheres; 13 females) with trOCD that underwent Nac/ALIC DBS were included. Mean age at surgery was (42.85 ± 9.09 years).

**Fig. 2:**
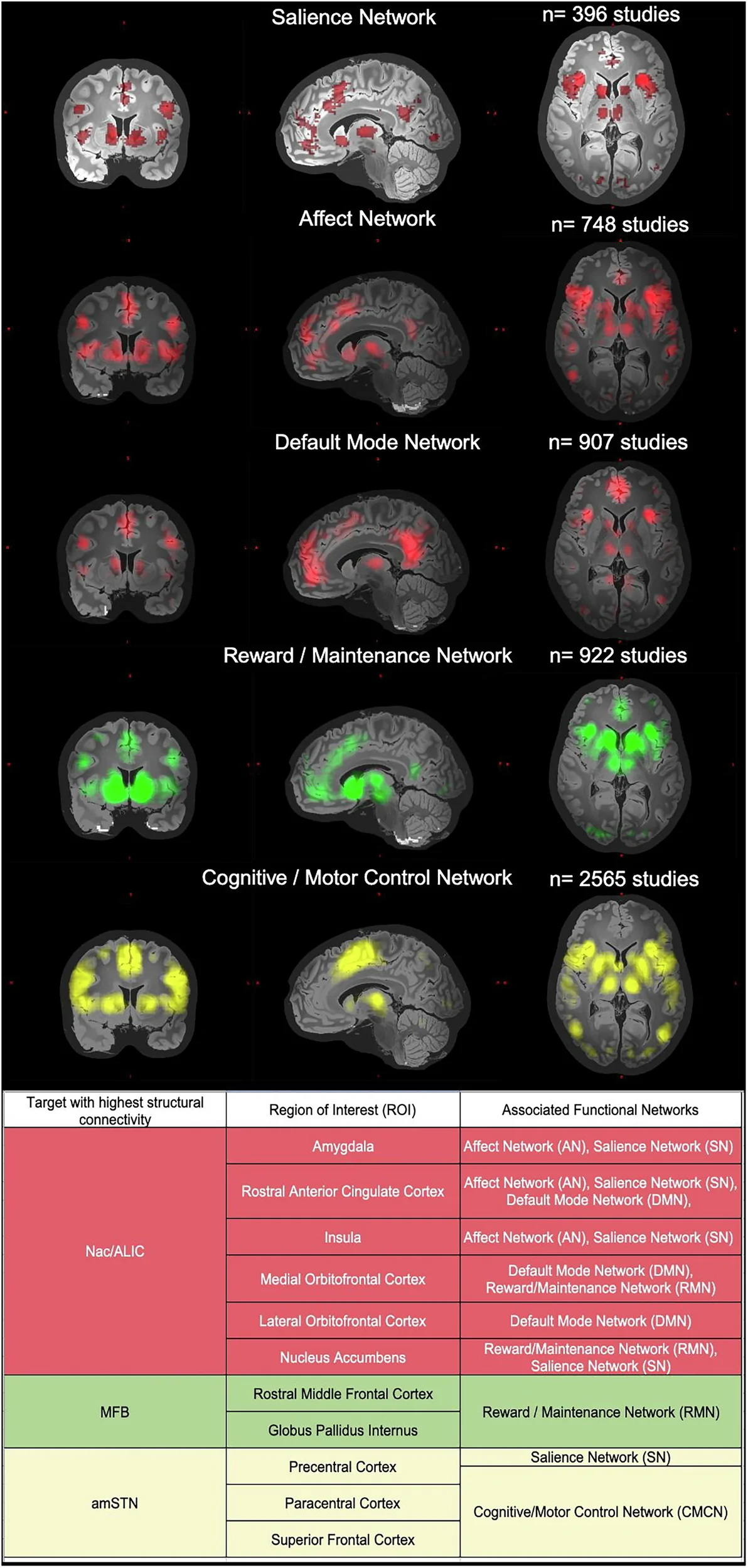
Distribution of regions of interest (ROIs) across five uniformity test meta-analysis functional brain networks involved in OCD. The Salience Network (SN), Reward/Maintenance Network (RMN), Affect Network (AN), Cognitive/Motor Control Network (CMCN), and Default Mode Network (DMN), and their strongest structural connectivity to one of three anatomical regions for trOCD DBS: the nucleus accumbens/anterior limb of the internal capsule (NAc/ALIC, red), the medial forebrain bundle (MFB, green), or the anteromedial subthalamic nucleus (amSTN, yellow). Each ROI is coloured based on its network affiliation, and the corresponding column indicates the DBS target with the highest streamline count in structural connectivity analyses. This pattern highlights target-specific structural affinity and suggests functional specificity of anatomical regions for modulating trOCD-relevant networks.

**Fig. 3:**
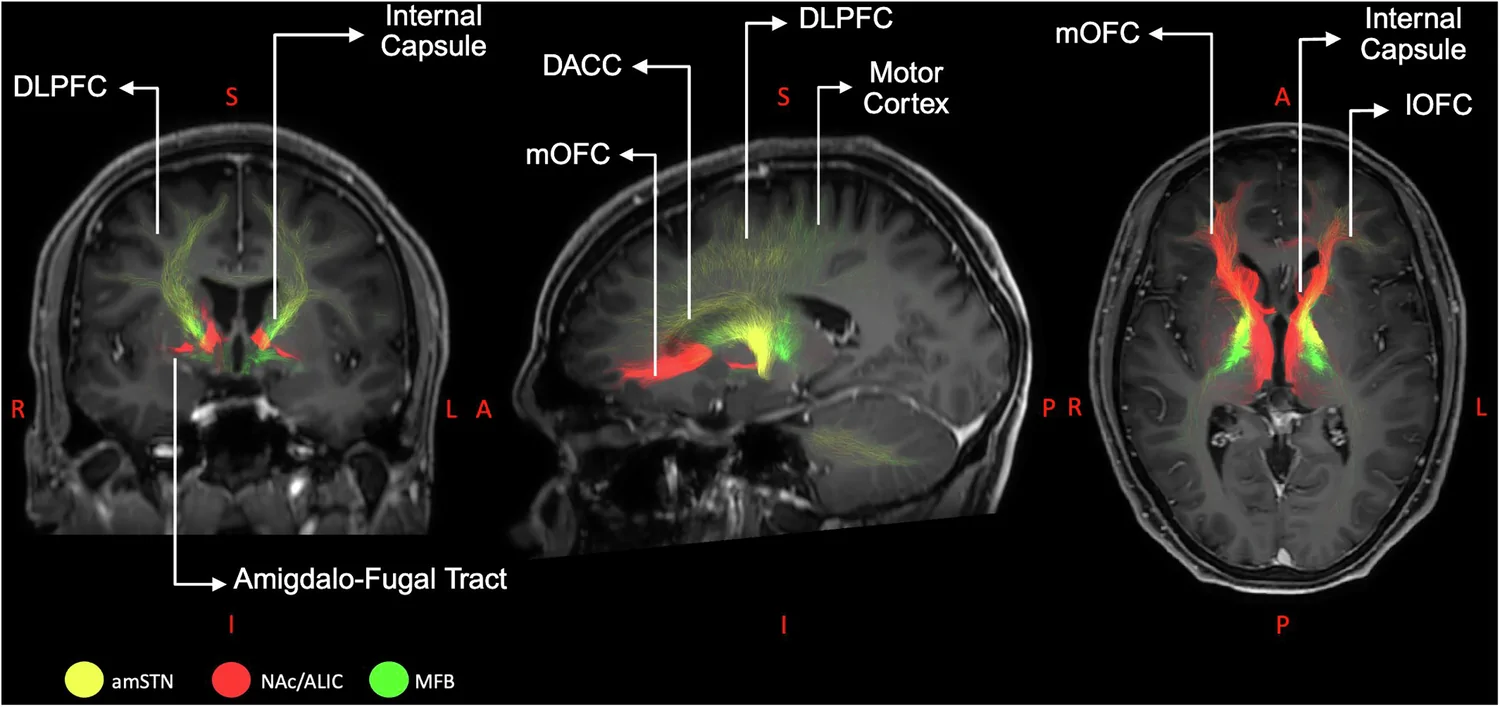
Qualitative analysis of structural connectivity in the amSTN, MFB and NAc/ALIC regions in 1 patient. Abbreviations: DACC = Dorsal Anterior Cingulate Cortex; DLPFC = Dorsolateral Prefrontal Cortex; mOFC = Medial Orbitofrontal Cortex; lOFC = Lateral Orbitofrontal Cortex.

Visual qualitative analysis of amSTN, MFB, and NAc/ALIC streamlines showed different structural connectivity profiles, represented as streamlines reaching different distant regions of the brain and correlating with meta-analysis results of the functional networks and in 1 representative patient (Figs. 2 and 3).

**Fig. 4:**
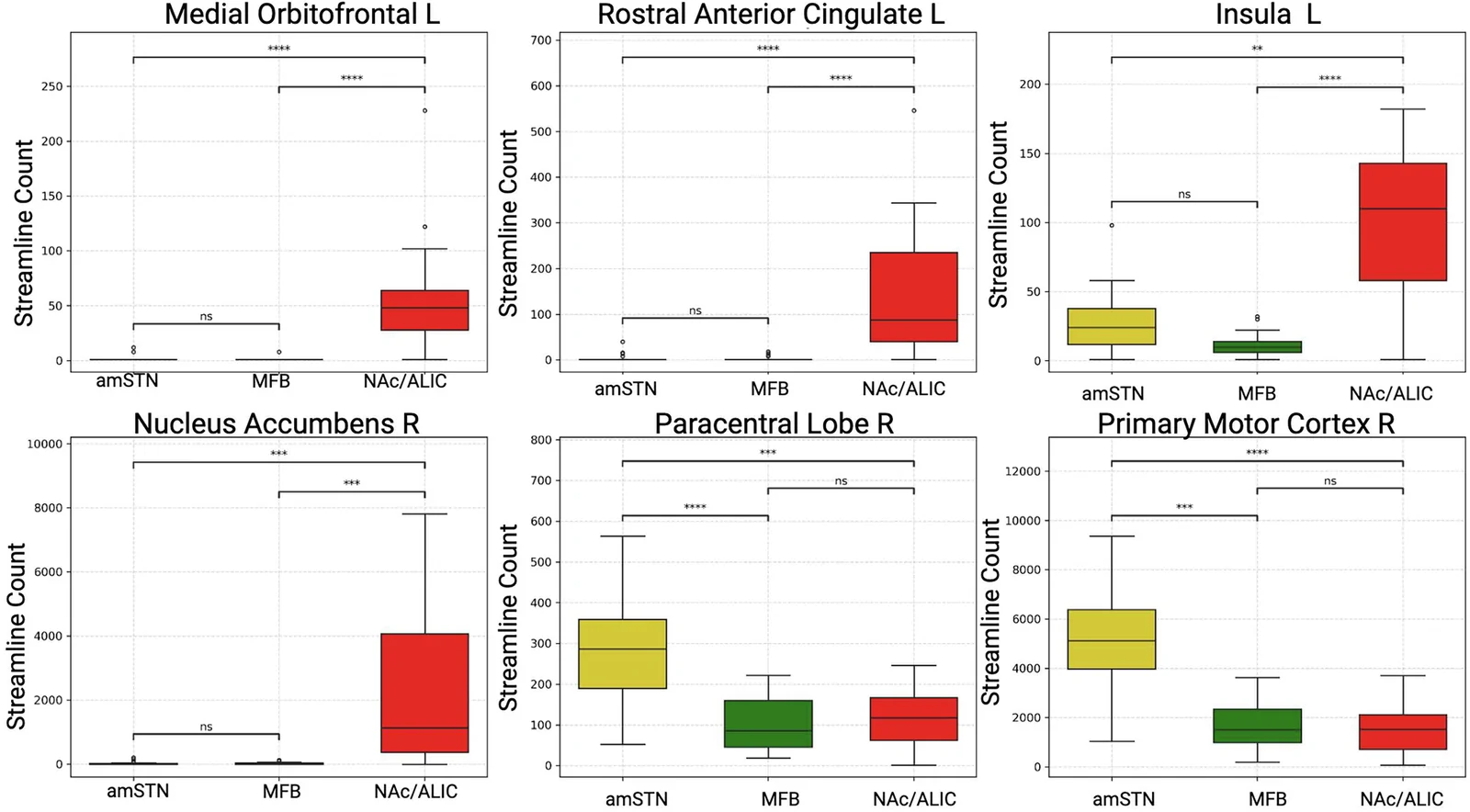
Streamline count between each trOCD DBS target (amSTN: yellow, MFB: green, NAc/ALIC: red) and key cortical and subcortical ROIs, grouped by functional sub-networks. Significant differences were observed in streamline counts from NAc/ALIC to limbic (e.g., medial orbitofrontal cortex, rostral anterior cingulate cortex, insula, and nucleus accumbens) and associative cortices, consistent with its connectivity to the affective and reward-related circuitry. The amSTN showed stronger connectivity to the paracentral lobe and the primary motor cortex, aligning with motor and cognitive control networks. MFB connectivity was intermediate across most ROIs. Asterisks denote significance levels (*p < 0.05, **p < 0.01, ***p < 0.001, ***p < *0.0001); ns = not significant)*.

Structural connectivity analyses revealed significant differences across anatomical regions for DBS targets in several structural and functional regions of interest. Notably, the NAc/ALIC target showed the highest mean streamline counts in fronto-limbic regions, including the medial orbitofrontal cortex (right: 155 ± 128.41; left: 56 ± 51.79), lateral orbitofrontal cortex (right: 158.84 ± 126.55; left: 253.11 ± 177.04), insula (right: 158 ± 124.73; left: 99 ± 52.82), amygdala (right: 341 ± 119; left: 94 ± 99) and rostral anterior cingulate cortex (right: 224.05 ± 271.13; left: 153.16 ± 144.68). These values considerably exceeded those observed in the MFB and amSTN groups. Post hoc comparisons confirmed statistically significant differences (all *p* < 0.001) between targets for these regions (Fig. 4).

**Fig. 5:**
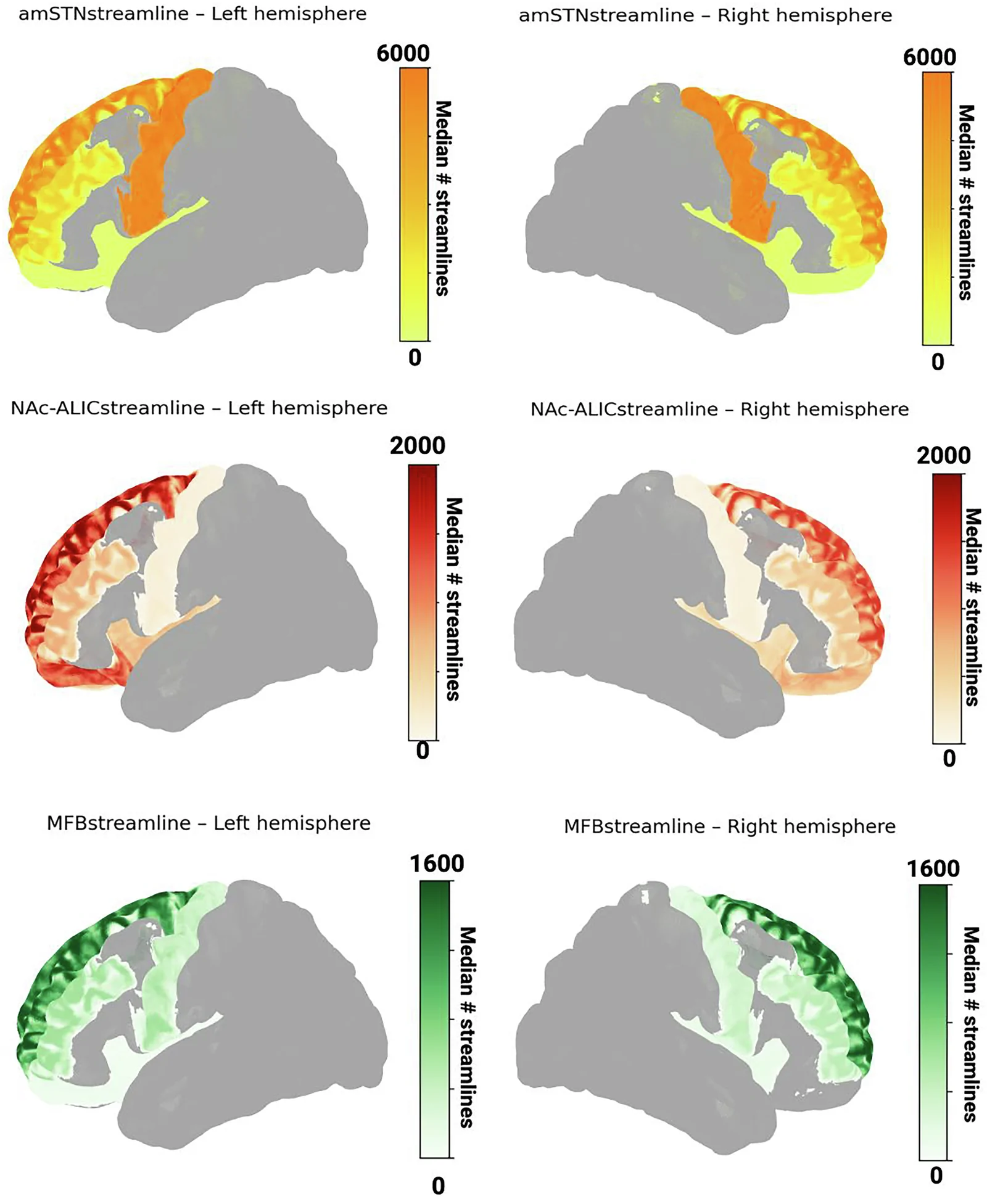
Distinct cortical connectivity profiles of three anatomical regions for trOCD. The amSTN shows the highest streamline density (up to 6000 streamlines) to motor-related areas, the NAc/ALIC (up to 2000 streamlines) primarily connects to frontolimbic regions involved in emotional regulation, and the MFB (up to 1600 streamlines) projects to reward-related areas. These profiles highlight the potential for symptom-specific neuromodulation.

In contrast, the amSTN group demonstrated increased structural connectivity with motor-associated areas such as the precentral gyrus (right: 5114.32 ± 2100.65; left: 1346.32 ± 1245.05), paracentral lobule (right: 45.58 ± 49.43; left:147.11 ± 127.55), and superior frontal gyrus (right: 850.95 ± 432.46; left: 599.33 ± 417.25). The MFB showed a predominant connectivity engaging the rostral middle frontal cortex (Brodman 8BL, 9 M, 9 P) (right: 96.12 ± 62.58; left: 121.88 ± 86.03) and pallidum (right: 1756.35 ± 973.25; left: 616.29 ± 572.81) (Fig. 4) (Fig. 5).

Kruskal-Wallis tests revealed statistically significant differences in streamline density and tract integrity metrics (FA, MD) among the three anatomical regions for DBS targets. FA values sampled along the Fp-Ct tract selection differed significantly across targets. Mean FA values were lower for NAc/ALIC tracts (right: 0.34 ± 0.04; left: 0.34 ± 0.04) than for amSTN tracts (right: 0.49 ± 0.05; left: 0.48 ± 0.05) and MFB tracts (right: 0.47 ± 0.06; left: 0.47 ± 0.06), with significant bilateral differences between NAc/ALIC and both comparator targets (*p* < 0.0001 for all comparisons).

MD values were similar across targets, with a small significant difference between NAc/ALIC and amSTN in the right hemisphere (*p* = 0.036)(Supplementary Figure 3).

## Discussion

### Summary of main findings

This study highlights that the three most commonly investigated anatomical regions for DBS targets for trOCD, the NAc/ALIC, the MFB, and the amSTN, present different patient-specific profiles of structural and functional connectivity networks involved in trOCD pathophysiology. Each anatomical region for DBS target showed a structural connectivity with large-scale cortico-subcortical functional networks associated with affect regulation, reward processing, or motor inhibition, respectively. Importantly, overlapping streamlines across all targets were observed in the ALIC [51], supporting a model in which DBS for trOCD efficacy arises from the modulation of partially convergent fronto-striatal, fronto-thalamic, mesolimbic, mesocortical, and cortico-subthalamic networks.

### Moving beyond the “Average Brain” mapping for individual patients

Traditional dMRI and fMRI analyses often depend on healthy population-based atlases or “average brain” templates to study at a group level. However, such approaches overlook the considerable variability in structural and functional brain organization between individuals [6, 15]. This lack of patient-specific resolution may have obscured biologically meaningful differences in previous group-level studies. Gratton et al. introduced a paradigm shift from population-averaged brain maps toward individualized functional neuroanatomy, a framework particularly relevant for precision-guided neuromodulation techniques such as DBS and rTMS for OCD treatments [11]. Similarly, previous research in functional connectivity demonstrated that, among patients with contamination or washing symptoms, clinical improvement correlates with connectivity between the ventral attention network and the medial prefrontal cortex [6].

Building on this evidence, our findings support the notion that patient-specific structural connectivity may capture critical network-level differences that may influence symptom-specific DBS outcomes based on structural and functional networks.

The connectivity analysis performed on this study were patient-specific at the level of diffusion data and T1 and T2 weighted MRI spatial transformation, but not at the level of anatomic surgical target selection. Although the predefined 2 mm radius spherical stereotactic ROIs representing clinically relexvant trOCD-DBS target regions were transformed into each patient´s native T1- and T2 weighted and b0 diffusion MRI spaces using subject-specific ANTs transformations, their centers were derived from standardised stereotactic coordinates rather than from surgical target selection performed independently for each patient´s anatomy. This strategy ensured equivalent spatial sampling volumes across targets and facilitated direct comparison of tractography derived structural connectivity estimates.

Our patient-specific tractography approach allowed estimation of relative, tractography derived structural connectivity, revealing how anatomical regions differentially engage cortico-subcortical structural and functional networks that may be linked to distinct OCD symptom dimensions. The present findings extend prior work based on normative connectomes by demonstrating that patient-specific tractography enables individualized estimation of tractography xderived structural connectivity and for sampling diffusion-derived metrics along target-specific streamline selections between predefined ROIs and each anatomical region serving as DBS target point [11]. This approach allowed us to characterize the structural connectivity profile of each target within the patient’s own neuroanatomical space, rather than relying on normative templates. In general, prior work is limited to a subset of normative connectome analysis. Our findings provide insights into target-specific modulation strategies, with a focus on probable hub regions of affective, salience, reward, and motor-inhibitory functional networks in patient-specific space. However, our results may not capture actual surgical targets relevant to clinical DBS prospective surgical planning. Future studies should integrate actual surgical targets, electrode localisation, stimulation parameters, electric-field modelling by volume of tissue activated, pathway activation models, and clinical outcomes to determine whether anatomy-guided target refinement improves the predictive value and translational relevance of these connectivity profiles.

### The role of patient-specific connectomics in DBS for trOCD

Rather than relying on a single standardised stereotactic coordinate (e.g., AC-PC or MNI space), we examined structural connectivity profiles among 13 anatomically defined ROIs (cortical, subcortical, and white-matter regions) across 22 trOCD patients. These ROIs were selected a priori based on their established role in OCD neurocircuitry and their cortical and subcortical projections involved in emotional regulation, motor inhibition, and reward processing [3, 47].

From a clinical perspective, these findings underscore the potential value of individualised DBS planning approaches. Target-specific structural connectivity profiles derived in patient space may help generate hypotheses about stimulation strategies tailored to specific symptom profiles. By integrating patient-specific tractography derived structural connectivity estimates with functional network models, the present study provides an exploratory framework for investigating how clinically relevant trOCD-DBS targets may relate to symptom-relevant neural circuits [6, 11].

Our results are consistent with previous studies linking the NAc/ALIC to frontolimbic regions involved in emotional regulation, cognitive control, and flexibility, particularly the medial orbitofrontal cortex, insula, and rostral anterior cingulate cortex [3, 5, 51–54]. Meyer et al. identified optimal stimulation sites (“sweet spots”) within the medial ventral capsule/ventral striatum, corresponding very close to the NAc/ALIC region, associated with significant clinical improvement in Y-BOCS scores [51]. Their study primarily aimed to refine targeting within a confined anatomical zone between the NAc and the medial ALIC. In contrast, the MFB exhibited connectivity with mesolimbic and reward-related regions, including the pallidum and rostral middle frontal cortex, while the amSTN showed preferential connections with motor-executive areas such as the precentral and paracentral gyri [12, 55]. Extending previous observations, our results indicate that the average OCD response tract may converge in dorsomedial prefrontal territories, including Brodmann areas 8BL, 9 M, and 9 P, with overlapping streamlines traversing the ALIC across all three targets [7, 51]. Together, these findings support the hypothesis thaxt DBS efficacy in trOCD arises from modulation of a partially convergent frontolimbic pathway, whose engagement may vary according to each patient’s symptom profile and structural connectivity [10, 12]. These observations may help generate hypotheses about how target choice relates to symptom-relevant networks. Nonetheless, larger multicentric studies are required to establish whether the symptom-informed strategy has clinical value.

Previous studies in OCD patients have reported increased fractional anisotropy (FA) in bilateral regions of the anterior limb of the internal capsule (ALIC), adjacent to the caudate body, along with decreased FA in the right anterior limb near the caudate head [3]. Additionally, lower mean diffusivity (MD) values have been observed in the body of the right cingulum and the left anterior cingulum compared with healthy controls [56]. Although our dataset exclusively includes patients with treatment-resistant OCD, we identified significant FA differences within the frontopontine-corticothalamic (Fp-Ct) tract across the NAc/ALIC, MFB, and amSTN targets. These results support the proof-of-concept that tractography-derived connectivity profiles of different stereotactic target ROIs representing clinically relevant trOCD-DBS target regions may intersect partially overlapping components of broader cortico-subcortical systems, including fronto-thalamic, mesocortical, mesolimbic, and hyperdirect cortico-subthalamic pathways. This may be useful to render the tracts in planning commercial software that uses FA and MD as thresholds for surgical planning. Such variability could reflect target-specific modulation profiles, potentially informing the refinement of personalized DBS strategies for optimizing therapeutic efficacy [55].

### Differential structural and functional connectivity of NAc/ALIC, MFB, and amSTN in trOCD

**Fig. 6:**
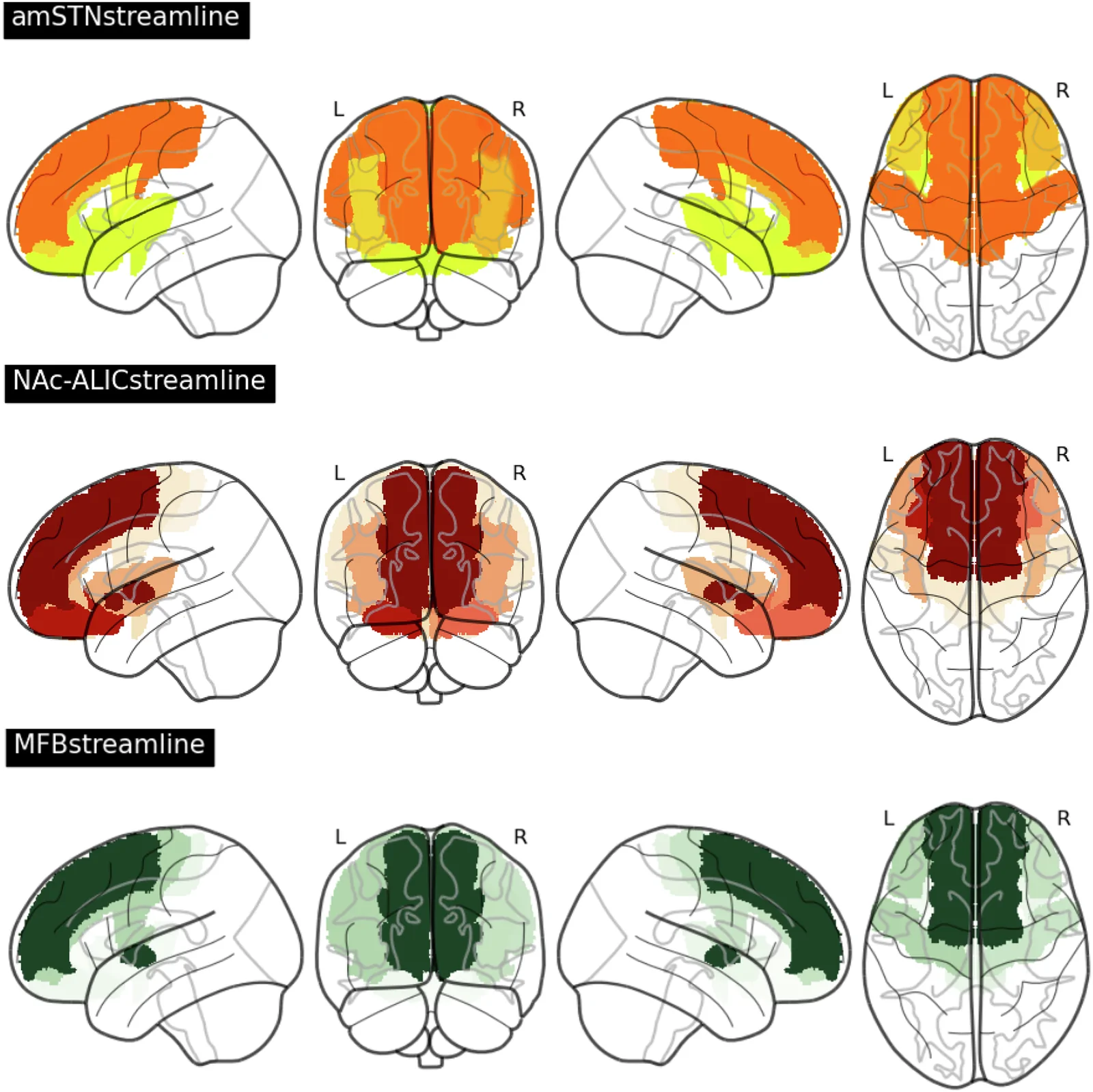
Cortical structural connectivity profiles of three anatomical regions for trOCD, based on patient-specific diffusion MRI tractography. The amSTN (yellow-top) projects mainly to sensorimotor areas involved in motor planning, such as the precentral and paracentral gyri. The NAc/ALIC (red-middle), shows strong connectivity with frontolimbic regions implicated in emotional regulation, including the medial and lateral orbitofrontal cortex, insula, and anterior cingulate. In contrast, the MFB (green-bottom) is primarily linked to reward and motivational circuits, projecting to the rostral middle frontal cortex and pallidum. These distinct profiles underscore each target’s potential for network-specific modulation in OCD.

To our knowledge this is the first research to evaluate, in parallel, the structural connectivity in patient-specific space of three primary anatomical regions used in trOCD DBS and to map in the resulting structural connectivity profiles onto five key functional networks underlying CSTC associated with OCD physiopathology: the reward/maintenance (RMN), salience (SN), affect (AN), cognitive/motor control (CMCN), and default mode (DMN) networks [47] (Fig. 6).

Our results reveal that the NAc/ALIC, amSTN, and MFB targets exhibit distinct profiles of structural connectivity across prefrontal-limbic and sensorimotor regions, which map onto functionally specialized cortical territories as shown in CSTC anatomical frameworks [5, 52]. Targeting of the NAc/ALIC target showed the most robust and widespread connectivity with fronto-limbic regions, particularly with the medial and lateral orbitofrontal cortex (BA 10, 11, 12), rostral anterior cingulate cortex (BA 24, 32), and insula, all of which reside within the ventromedial and paralimbic zones of the frontal lobe. The structural engagement of these regions positions the NAc/ALIC as a compelling target for modulating the AN and RMN in OCD, particularly in patients presenting affection regulation, mood stability, ritualistic behaviours driven by emotional dysregulation and compulsivity [2, 9, 52–54].

In contrast, the amSTN exhibited prominent connectivity to premotor and dorsolateral prefrontal areas (BA 8-9), both of which are involved in sensorimotor and attentional integration, core components of the CMCN, and may further contribute to alleviating rigidity and hyper-responsiveness to intrusive thoughts [12, 55]. These connections suggest the potential utility of amSTN DBS for patients with pronounced motor compulsions or cognitive inflexibility, motor rituals, or cognitive rigidity.

The MFB demonstrated selective structural engagement of the rostral middle frontal cortex and ventral pallidum, regions tightly linked to motivation, reward expectation, and drive [10]. Its limited involvement with motor or associative frontal cortices supports a more targeted modulation profile (potentially reducing cognitive side effects in specific clinical subtypes, attenuating the subcortical interpretation of obsession salience). Because of its functional relevance in the RMN, these target may be optimal for DBS patients with anhedonia, lack of motivation, or obsessive beliefs centered around reward prediction errors [10]. This pattern aligns with its acknowledged role in attenuating the salience of intrusive obsessive thoughts [5, 52, 54]. The fact that MFB projections did not significantly involve motor or associative frontal regions indicates a comparatively selective structural connectivity profile. Whether these patterns relate to different clinical or side-effect outcomes compared with the amSTN stimulation cannot be inferred from the present tractography derived structural connectivity analysis and requires prospective, outcome-linked validation.

Our findings emphasize the importance of considering the specific structural connectivity profiles when selecting a target for DBS in OCD patients, which is also crucial when using FA values as a threshold for tractography planning of these targets in trOCD DBS. However, FA and MD are diffusion derived metrics with limited biological specificity. Differences in these metrics across target-specific streamline selections may reflect several factors, including anatomical location, local tissue composition, FODs, fiber crossing regions, partial volume effects, and voxel-level averaging [57]. These measures should therefore be interpreted descriptively and should not be considered direct indicators of white-matter integrity, fiber coherence, axonal density, myelination, or cellular microstructure [58].

The reconstructed pathways should not be reduced to a single fronto-striatal model. Rather, the target regions may intersect partially overlapping components of broader cortico-subcortical systems, including fronto-thalamic, mesocortical, mesolimbic, and hyperdirect cortico-subthalamic pathways. These findings should be considered within the broader framework of circuit-based neuromodulation for OCD, where DBS is increasingly conceptualized as modulation of distributed cortico-subcortical networks rather than isolated anatomical targets [59]. Patient-specific tractography may therefore provide an exploratory approach for characterising how different stereotactic target regions engage partially overlapping but distinct structural networks. The observed connectivity profiles may provide a framework for generating hypotheses about symptom-specific networks for DBS surgical planning strategies; however, the present study did not directly test associations between OCD symptom dimensions and target-specific connectivity. This relationship therefore remains theoretical and requires validation in future cohorts with dimensional phenotyping and longitudinal outcomes.

### Limitations

Several limitations should be considered when interpreting these findings. First, the absence of a non-OCD control group limits our ability to characterise structural-connectivity profiles that are specific to OCD. Second, detailed symptom-dimension data were not available for all clinically relevant trOCD-DBS target regions, precluding a direct symptom-connectivity analysis. Third, the modest sample size limits statistical power and generalisability, and hemisphere-level observations from the same patient are not fully independent; the findings should therefore be regarded as exploratory and hypothesis-generating. This limited cohort size also reflects broader challenges in trOCD-DBS research, including the rarity of DBS implantation, restricted access to specialised care, regulatory and reimbursement barriers, and the absence of prospective international registries.

A further consideration is that all patients underwent NAc/ALIC DBS, and the cross-target comparison was performed on preoperative MRI using predefined stereotactic ROIs rather than implanted electrodes. The MFB and amSTN analyses are therefore not based on patients implanted in those targets and should not be interpreted as target-specific clinical-outcome profiles; they represent structural-connectivity estimates derived from standardised 2 mm radius spherical ROIs propagated into each participant’s native diffusion space.

Finally, probabilistic tractography has inherent limitations, including the reconstruction of false-positive streamlines and anatomically implausible pathways, particularly in regions of crossing fibers such as the internal capsule [57, 60]. Reconstructed streamlines should therefore be interpreted as computational estimates of probable white-matter trajectories rather than definitive proof of axonal connectivity, and a higher streamline count for a given target ROI should not be read as an exact estimate of axon number or as evidence of stronger physiological modulation. Accordingly, the present results should be regarded as relative, tractography-derived differences in streamline representation obtained within a consistent patient-specific pipeline [60]. Similarly, FA and MD should not be interpreted purely as indices of white-matter integrity or cellular density, as both metrics have a limited ability to distinguish the specific microscopic factors influencing their values, partly owing to the relatively large voxel size in diffusion imaging [57, 58].

Future studies should combine validated clinical scores for OCD subtype phenotyping, electrode localisation, stimulation modelling, and longitudinal outcomes to determine whether specific symptom clusters map onto individualised connectivity profiles.

## Conclusions

The present study evaluates patient-specific tractography derived structural-connectivity profiles associated with standardised stereotactic target ROIs mapped into native space, rather than fully individualized surgical targets. This study demonstrates that three commonly used anatomical regions serving as targets for DBS in trOCD patients, such as the NAc/ALIC, MFB, and amSTN, may engage different structural connectivity profiles, each aligning with specific functional networks. These findings support the hypothesis that, although a partially shared structural connectivity may mediate symptomatic improvement, each tROCD DBS target region may modulate unique cortico-subcortical circuits. The differential connectivity profiles observed here may help explain the variability in clinical outcomes across anatomical regions for DBS in trOCD.

In the future, DBS planning could incorporate patient-specific symptomatology based on this connectomic framework to refine target selection and programming strategies. These exploratory findings motivate prospective studies, to test whether network-based, symptom-informed target selection has clinical value across affective dysregulation, intrusive thoughts, and motor compulsions. Ultimately, this strategy holds promise for advancing precision neuromodulation in trOCD, improving both efficacy and individualization of therapy. Future multicentric studies are required to validate these structural connectivity profiles and further optimize target selection for symptom-specific DBS planning.

## Supplementary information

Supplementary Material

## Data Availability

Due to ethical and institutional data protection policies, direct public access is restricted. However, data may be available for collaborative research purposes upon reasonable request and pending approval by the University Hospital of Cologne.
