# Supplementary material for: Patient-specific structural connectomic differences of deep brain stimulation targets in treatment-resistant obsessive-compulsive disorder patients
Source: Transl Psychiatry. 2026 Sep 22;16:478. doi: 10.1038/s41398-026-04441-4 (PMC13598114; doi:10.1038/s41398-026-04441-4)
Supplement: Supplementary file 1 [file 41398_2026_4441_MOESM1_ESM.docx]

**Supplementary Materials**

**S1. Patient Cohort**

We retrospectively analysed patient-specific native space structural connectivity in a single-center cohort, representing each clinically relevant trOCD-DBS target as a standardised 2-mm-radius spherical ROI. We retrospectively analysed structural and diffusion-weighted imaging (DWI) data from 22 trOCD patients (44 brain hemispheres), all of whom underwent DBS surgery between 2016 and 2025 at the Department of Stereotactic and Functional Neurosurgery, University Hospital Cologne, Germany. Inclusion criteria were:

A: A confirmed DSM-5 diagnosis of OCD by a psychiatrist;

B: Non-response to at least two selective serotonin reuptake inhibitors (SSRIs), cognitive behavioural therapy, and augmentation with antipsychotics;

C: Eligibility for DBS based on institutional guidelines.

Exclusion criteria included a history of neurological disease, active substance abuse, and incomplete MRI sequence data.

**S2. Ethical Considerations**

All procedures were approved by the Ethics Committee of the University of Cologne (DRKS00005316) and adhered to the principles of the Declaration of Helsinki. All patients provided written informed consent.

**S3. Imaging Acquisition**

MRI was performed using a 3T system (Philips Ingenia, Eindhoven, Netherlands). T1- and T2-weighted structural images were acquired with 0.5 x 0.5 x 1 mm³ resolution. T1 with TR/TE: 9.75/4.86 ms, and T2 with TR/TE: 3000/80 ms. Diffusion-weighted images (dMRI) included 40 gradient directions (b = 1,000 s/mm²) with AP phase encoded direction, 1 b0 with AP phase encode, and 1 b0 with PA phase encode. dMRI with TR/TE: 8218/103 ms. dMRI resolution was 2 mm isotropic. Each scanning session lasted approximately 20 minutes.

**S4. MRI data pre-processing**

In our study, the preprocessing of MRI data was primarily conducted using MRtrix3 software commands, supplemented by tools from Freesurfer (version 7.4.1), FSL (version 6.07.18), and Advanced Normalization Tools (ANTs) (v3.26.3)(1,2,3,4). To enhance the quality of the dMRI data, we used the *dwidenoise* command for estimating the spatially varying noise map (5,6). Gibb’s ringing artifacts were mitigated using the *mrdegibbs* command (7). We employed the *dwifslpreproc* command, which integrates FSL's *eddy* and *topup* tools to address motion artifacts, B0 field inhomogeneity, eddy currents, and distortions (3,8,9). Subsequent bias field corrections were made with the *dwibiascorrect* command to refine brain mask estimations (10).

For delineating the brain structure more accurately, we generated a binary brain mask using the *dwi2mask* command, ensuring the biological plausibility of streamline generation (1). The tissue response functions for white matter (WM), as well as gray matter (GM) and cerebrospinal fluid (CSF), were derived using the *dwi2response* command with the *Dhollander* algorithm (11,12,13). *Single-Shell 3-Tissue constrained spherical deconvolution* was performed to obtain WM-like fiber orientation distributions (FODs) as well as GM-like and CSF-like compartments in all voxels (14,15,16).

Global intensity normalization was performed using the *mtnormalise* command to ensure comparability of FODs across groups (1). To prepare for streamline seeding, we first conducted skull stripping with FSL's *bet* function, followed by tissue segmentation using Freesurfer's *recon-all* command on the T1 images (2,17). Brain tissue boundaries were further delineated with the *5ttgen* function (1). Spatial alignment between the T1 images and each patient's native dMRI space was achieved through linear registration using the *antsRegistration* function, with the CSF volume extracted via the *fslsplit* function from the dMRI b0 images (4,18). Each step of co-registration and normalization was meticulously reviewed and adjusted as needed to ensure accuracy. Upon obtaining satisfactory alignment, the inverse spatial transformation was applied, by means of *antsApplyTransforms* function, using an affine registration to align the T1 to the dMRI space (4). The final stage involved generating a mask image at the grey matter-white matter interface (GMWMI) with the *5ttgmwi* command, providing an optimal starting point for seeding streamlines (1).

**S5. Anatomical constrained tractography**

Whole-brain probabilistic tractography was performed for each subject using MRtrix3 (v 3.0.8). Anatomically constrained tractography was used to improve the anatomical plausibility of streamline termination at the GM-WM interface. For each patient, 10 million streamlines were initially selected in the whole brain. Streamline length was restricted from 10 to 250 mm, the minimum FOD amplitude threshold was set to 0.05, and the maximum angular deviation was set to 25 degrees.

Afterwards, we reduced the number of streamlines to 1 million using Spherical deconvolution informed filtering of Tractograms (SIFT) (19,20). SIFT was implemented to reduce known streamline reconstruction biases and to improve the correspondence between streamline density and fiber density estimated from the FODs. SIFT was used as part of the processing pipeline rather than as an experimental test of this analysis.

**S6. Effect of SIFT filtering on target-specific streamline counts**

This supplementary analysis assessed how SIFT filtering to 1 million streamlines affected target-specific streamline counts relative to the original 10 million whole-brain tractogram in one patient. No formal comparison with unfiltered 10 million whole-brain tractograms was performed. The analysis was intended as a methodological sensitivity assessment of SIFT filtering on target-specific streamline counts, not as a comparison of tractography algorithms or as evidence of absolute anatomical connectivity or clinical relevance. See **Supplementary Figure 1.**

Target-specific tractograms were extracted from both the original and SIFT-filtered whole-brain tractograms. Streamline counts were quantified in native b0 diffusion space and rechecked after transformation to native T1-weighted and MNI152NLin2009b spaces to verify preservation of tractogram size across coordinate transformations.

Across the six target ROIs, the original tractogram yielded a mean of 44,402 target-specific streamlines, whereas the SIFT-filtered tractogram yielded a mean of 3,723 streamlines. Pooled across targets, SIFT retained 8.38% of target-specific streamlines. Across individual targets, mean retention was 14.47%, with a median of 7.96% and a range of 5.39% to 44.12%. Thus, SIFT markedly reduced absolute streamline counts while preserving non-zero target-specific streamline subsets across all analysed ROIs. These results should be interpreted as tractography derived methodological metrics, not as measures of axonal number, fiber density, or absolute structural connectivity.

### **S7. Transformation of MNI-space DBS target ROIs into patient-specific diffusion space**

For the comparative patient-specific structural connectivity analysis, each clinically relevant trOCD-DBS target was modelled as a binary standardised spherical ROI with a radius of 2 mm in MNI152NLin2009bAsym template space. Bilateral ROIs were generated separately for the medial forebrain bundle (MFB), nucleus accumbens/anterior limb of the internal capsule (NAc/ALIC), and anteromedial subthalamic nucleus (amSTN). ROI centers were defined from predefined stereotactic coordinates in MNI152NLin2009bAsym space, based on a high-resolution 7T ex vivo T1-weighted MRI volume and standardised using the tenths based stereotactic method referenced to the BigBrain AC-PC line (15,22,25). The AC-PC distance in BigBrain was 25.615 mm, corresponding to a stereotactic tenth value of 2.5615 mm. The anterior commissure coordinate was visually refined to x = -0.197928, y = 1.66628, and z = -3.7029 mm (22). The complete list of target coordinates is provided in **Supplementary Table 1.**

The MNI-space ROIs were subsequently transformed into each participant’s native diffusion space using a sequential registration workflow. Native T1-weighted images were first corrected for intensity inhomogeneity using N4 bias-field correction. A nonlinear registration between native T1-weighted space and the adult MNI152NLin2009bAsym template was then performed using Advanced Normalization Tools (ANTs). The forward registration from native T1-weighted image to MNI space comprised four sequential optimisation stages: rigid-body registration, affine registration, a global symmetric normalisation (SyN) deformable registration, and a second SyN refinement constrained by a subcortical mask. The rigid and affine stages corrected for differences in head position, orientation, scale, and global brain geometry. The first SyN stage modelled nonlinear anatomical variability across the whole brain, whereas the second masked SyN stage preferentially refined the alignment of DBS-relevant subcortical structures.

Because the estimated ANTs transformations mapped native T1-weighted images to MNI space, their inverse transforms were applied to propagate the predefined ROIs into each participant’s native T1-weighted image. Native b0 images were subsequently registered to the corresponding T1-weighted images using rigid and affine transformations based on mutual information. The inverse b0-to-T1 affine transformation was then applied to map the ROIs from native T1-weighted space into native b0 space.

For quality-control, ROI propagation was generated both as a two-step workflow (MNI to native T1 to native b0) and as a single concatenated transformation (MNI to native b0). The single-step transformation was used for the final patient-specific ROIs because it minimised repeated interpolation. Nearest-neighbour interpolation was applied to all binary ROI masks to preserve label integrity, whereas linear interpolation was used for anatomical quality-control images. Each transformed ROI was re-binarised after resampling, and its final voxel count and physical volume were recorded to identify empty masks, excessive fragmentation, or substantial volume changes. All transformed ROIs were visually inspected in both native T1-weighted and b0 spaces before tractography-based streamline extraction using tckedit.

The resulting ROIs were used as standardised stereotactic sampling regions for patient-specific tractography-derived structural connectivity analysis. For each target, probabilistic streamlines intersecting the native-space ROI were extracted, and tract-density maps and tractography derived connectivity metrics were calculated. These spherical ROIs should not be interpreted as complete anatomical segmentations or as biophysically modelled volumes of tissue activated, because stimulation parameters, electrode-contact configurations, tissue conductivity, and electric-field modelling were not incorporated into their definition. Representative examples of patient-specific localisation of standardised spherical ROIs representing trOCD-DBS targets in native anatomical space are shown in the main manuscript, **Figures 1A and 1B.**

**S8. Cross-Platform Registration and Tract Analysis**

To align MRtrix3 tractograms with DSI-Studio (v2023), FA maps were used as reference and co-registered using ANTs antsRegistration. Tractograms were transformed into DSI-Studio space and connectivity matrices generated using anatomical parcellations (26,27,28). The HCP842_tractography atlas guided white matter bundle definitions (28). The CerebrA atlas guided the a-priori ROIs streamline count (29). Metrics included a-priori ROI streamline count, FA, and MD.

**S9. Structural Connectivity and Tractometry**

We calculated streamline count between each brain hemisphere NAc/ALIC, amSTN and MFB tractogram and a-priori ROIs (amygdala, accumbens, medial orbitofrontal cortex, lateral orbitofrontal cortex, rostral anterior cingulate, ínsula, rostral middle frontal, pallidum, precentral, paracentral, superior frontal) and meso-limbic fiber bundle, the frontopontine-thalamocortical tracts (Fp-Ct). Streamline count from ROIs that passed through the NAc/ALIC, amSTN, and MFB were quantified. Global FA and MD values were averaged along each tract and compared across targets. For FA and MD boxplots see **Supplementary Figures 2 and 3.**

**References:**

1. Tournier JD, Smith R, Raffelt D, Tabbara R, Dhollander T, Pietsch M, et al. MRtrix3: a fast, flexible and open software framework for medical image processing and visualisation. Neuroimage. 2019;202:116137. doi:10.1016/j.neuroimage.2019.116137.

2. Fischl B. FreeSurfer. Neuroimage. 2012;62(2):774-81. doi:10.1016/j.neuroimage.2012.01.021.

3. Smith SM, Jenkinson M, Woolrich MW, Beckmann CF, Behrens TEJ, Johansen-Berg H, et al. Advances in functional and structural MR image analysis and implementation as FSL. Neuroimage. 2004;23 Suppl 1:S208-19. doi:10.1016/j.neuroimage.2004.07.051.

4. Avants B, Tustison NJ, Song G. Advanced Normalization Tools: V1.0. Insight J. 2009. doi:10.54294/uvnhin.

5. Veraart J, Fieremans E, Novikov DS. Diffusion MRI noise mapping using random matrix theory. Magn Reson Med. 2016;76(5):1582-93. doi:10.1002/mrm.26059.

6. Veraart J, Novikov DS, Christiaens D, Ades-Aron B, Sijbers J, Fieremans E. Denoising of diffusion MRI using random matrix theory. Neuroimage. 2016;142:394-406. doi:10.1016/j.neuroimage.2016.08.016.

7. Kellner E, Dhital B, Kiselev VG, Reisert M. Gibbs-ringing artifact removal based on local subvoxel-shifts. Magn Reson Med. 2016;76(5):1574-81. doi:10.1002/mrm.26054.

8. Andersson JLR, Skare S, Ashburner J. How to correct susceptibility distortions in spin-echo echo-planar images: application to diffusion tensor imaging. Neuroimage. 2003;20(2):870-88. doi:10.1016/S1053-8119(03)00336-7.

9. Andersson JLR, Sotiropoulos SN. An integrated approach to correction for off-resonance effects and subject movement in diffusion MR imaging. Neuroimage. 2016;125:1063-78. doi:10.1016/j.neuroimage.2015.10.019.

10. Tustison NJ, Avants BB, Cook PA, Zheng Y, Egan A, Yushkevich PA, et al. N4ITK: improved N3 bias correction. IEEE Trans Med Imaging. 2010;29(6):1310-20. doi:10.1109/TMI.2010.2046908.

11. Dhollander T, Connelly A. A novel iterative approach to reap the benefits of multi-tissue CSD from just single-shell (+ b=0) diffusion MRI data. In: Proceedings of the International Society for Magnetic Resonance in Medicine; 2016; Singapore. p. 3010.

12. Dhollander T, Raffelt D, Connelly A. Unsupervised 3-tissue response function estimation from single-shell or multi-shell diffusion MR data without a co-registered T1 image. Presented at: ISMRM Workshop on Breaking the Barriers of Diffusion MRI; 2016; Lisbon, Portugal.

13. Dhollander T, Raffelt D, Connelly A. Accuracy of response function estimation algorithms for 3-tissue spherical deconvolution of diverse quality diffusion MRI data. In: Proceedings of the International Society for Magnetic Resonance in Medicine; 2018. p. 555-65.

14. Dhollander T, Mito R, Raffelt D, Connelly A. Improved white matter response function estimation for 3-tissue constrained spherical deconvolution. In: Proceedings of the International Society for Magnetic Resonance in Medicine; 2019. p. 555-65.

15. Aerts H, Dhollander T, Marinazzo D. Evaluating the performance of 3-tissue constrained spherical deconvolution pipelines for within-tumor tractography. bioRxiv. 2019. doi:10.1101/629873.

16. Calamuneri A, Arrigo A, Mormina E, Milardi D, Cacciola A, Chillemi G, et al. White matter tissue quantification at low b-values within constrained spherical deconvolution framework. Front Neurol. 2018;9:716. doi:10.3389/fneur.2018.00716.

17. Popescu V, Battaglini M, Hoogstrate WS, Verfaillie SCJ, Sluimer IC, van Schijndel RA, et al. Optimizing parameter choice for FSL-Brain Extraction Tool (BET) on 3D T1 images in multiple sclerosis. Neuroimage. 2012;61(4):1484-94. doi:10.1016/j.neuroimage.2012.03.074.

18. Jenkinson M, Beckmann CF, Behrens TEJ, Woolrich MW, Smith SM. FSL. Neuroimage. 2012;62(2):782-90. doi:10.1016/j.neuroimage.2011.09.015.

19. Smith RE, Tournier JD, Calamante F, Connelly A. Anatomically-constrained tractography: improved diffusion MRI streamlines tractography through effective use of anatomical information. Neuroimage. 2012;62(3):1924-38. doi:10.1016/j.neuroimage.2012.06.005.

20. Smith RE, Tournier JD, Calamante F, Connelly A. SIFT: spherical-deconvolution informed filtering of tractograms. Neuroimage. 2013;67:298-312. doi:10.1016/j.neuroimage.2012.11.049.

21. Fonov V, Evans A, McKinstry R, Almli C, Collins D. Unbiased nonlinear average age-appropriate brain templates from birth to adulthood. Neuroimage. 2009;47 Suppl 1:S102. doi:10.1016/S1053-8119(09)70884-5.

22. Edlow BL, Mareyam A, Horn A, Polimeni JR, Witzel T, Tisdall MD, et al. 7 Tesla MRI of the ex vivo human brain at 100 micron resolution. Sci Data. 2019;6(1):244. doi:10.1038/s41597-019-0254-8.

23. Coenen VA, Schumacher LV, Kaller C, Schlaepfer TE, Reinacher PC, Egger K, et al. Anatomy of the human medial forebrain bundle. Neuroimage Clin. 2018;18:770-83.

24. Mallet L, Polosan M, Jaafari N, Baup N, Welter ML, Fontaine D, et al. Subthalamic nucleus stimulation in severe obsessive-compulsive disorder. N Engl J Med. 2008;359(20):2121-34.

25. Márquez-Franco R, Concha L, García-Gomar MG, Carrillo-Ruíz JD, Loução R, Barbe MT et al. Validation of tenths stereotactic coordinates method using probabilistic tractography of the ansa lenticularis in Parkinson’s disease patients. World Neurosurg 2024;191:e235-45.

26. Yeh FC, Badre D, Verstynen T. Connectometry: a statistical approach harnessing the analytical potential of the local connectome. Neuroimage. 2016;125:162-71. doi:10.1016/j.neuroimage.2015.10.053.

26. Huang CC, Rolls ET, Feng J, Lin CP. An extended Human Connectome Project multimodal parcellation atlas of the human cortex and subcortical areas. Brain Struct Funct. 2022;227(3):763-78. doi:10.1007/s00429-021-02421-6.

27. Yeh FC, Panesar S, Fernandes D, Meola A, Yoshino M, Fernandez-Miranda JC, et al. Population-averaged atlas of the macroscale human structural connectome and its network topology. Neuroimage. 2018;178:57-68. doi:10.1016/j.neuroimage.2018.05.027.

28. Yeh FC. Population-based tract-to-region connectome of the human brain and its hierarchical topology. Nat Commun. 2022;13(1):4933. doi:10.1038/s41467-022-32595-4.

29. Manera AL, Dadar M, Fonov V, Collins DL. CerebrA: registration and correction of Mindboggle-101 atlas. Sci Data 2020;7(1):237.

**Figures**

**Supplementary Figure 1. Original and SIFT-filtered tractograms.**

Coronal, sagittal, and axial views show the original 10-million-streamline tractogram and the 1 million SIFT tractogram overlaid on native anatomical MRI. Full and cropped views are provided to aid inspection of target relevant streamlines. SIFT reduced streamline density while preserving the overall spatial organisation of the reconstructed trajectories. The figure is intended for quality control and methodological transparency, not as direct anatomical proof of axonal connectivity. ROIs are colour-coded as follows: amSTN, yellow; NAc/ALIC, red; and MFB, green.

**Supplementary Figure 2.** **Boxplots illustrate the distribution of Fractional Anisotropy (FA)**. The FA values of the Frontopontine-Thalamocortical (Fp-Ct) tracts for the right (FA_R) and left (FA_L) hemispheres of the anteromedial subthalamic nucleus (amSTN, yellow), medial forebrain bundle (MFB, green), and nucleus accumbens/anterior limb of the internal capsule (NAc/ALIC, red). Significant differences were observed between NAc/ALIC and both amSTN and MFB in both hemispheres, indicating lower FA values in the NAc/ALIC tracts. No significant differences were observed between amSTN and MFB. ***p < 0.001, ****p < 0.0001, ns = non-significant.

**Supplementary Figure 3. Boxplots illustrate the distribution of mean diffusivity (MD)**. The MD values (mm²/s) for streamlines associated with each DBS target anteromedial subthalamic nucleus (amSTN, yellow), medial forebrain bundle (MFB, green), and nucleus accumbens/anterior limb of the internal capsule (NAc/ALIC, red) in the right (MD_R, left panel) and left (MD_L, right panel) hemispheres. No statistically significant differences were observed between targets (ns: not significant) in either hemisphere, as determined by Kruskal-Wallis test followed by post hoc pairwise comparisons. Boxes represent interquartile ranges (IQR), with the median indicated by a horizontal line; whiskers indicate 1.5× IQR, and outliers are shown as individual points.

**Tables**

| **Target region** | **Hemisphere** | **X (mm)** | **Y (mm)** | **Z (mm)** |
| --- | --- | --- | --- | --- |
| MFB | Right | 6.90071 | -13.6624 | -11.0277 |
| MFB | Left | -6.1823 | -14.1487 | -11.2425 |
| NAc/ALIC | Right | 10.5625 | 8.56184 | -2.62849 |
| NAc/ALIC | Left | -10.4325 | 8.45619 | -2.62849 |
| amSTN | Right | 10.9656 | -12.7153 | -6.53569 |
| amSTN | Left | -10.9656 | -12.7153 | -6.53569 |

**Supplementary Table 1. Predefined stereotactic coordinates for standardized DBS target ROIs in trOCD.**

Predefined stereotactic coordinates for the MFB (23), amSTN (24), and NAc/ALIC were mapped onto a 7T BigBrain T1-weighted image with 100-micron resolution and standardised using the tenths stereotactic coordinates method referenced to the BigBrain AC-PC line (22,25). Standardised spherical ROIs were generated in the Big Brain MNI152NLin2009bAsym space and subsequently propagated into each participant’s native diffusion space (25). The NAc/ALIC coordinates were based on our center’s stereotactic targeting approach, approximately 8 mm lateral to the midline, 5 mm anterior to the anterior commissure, and 4 mm below the intercommissural line, with electrode placement intended to cover the NAc, NAc/ALIC transition, and ALIC, 2 mm from the diagonal band of Broca in the coronal plane. These ROIs should be interpreted as standardised stereotactic sampling regions rather than complete anatomical segmentations or biophysically modelled volumes of tissue activated. Abbreviations: ALIC, anterior limb of the internal capsule; amSTN, anteromedial subthalamic nucleus; DBS, deep brain stimulation; MFB, medial forebrain bundle; NAc, nucleus accumbens; ROI, region of interest; trOCD, treatment-resistant obsessive-compulsive disorder.
